# Comparative assessment of performance and genome dependence among phylogenetic profiling methods

**DOI:** 10.1186/1471-2105-7-420

**Published:** 2006-09-27

**Authors:** Evan S Snitkin, Adam M Gustafson, Joseph Mellor, Jie Wu, Charles DeLisi

**Affiliations:** 1 Bioinformatics Graduate Program, Boston University, Boston, USA; 2 Department of Biomedical Engineering, Boston University, Boston, USA

## Abstract

**Background:**

The rapidly increasing speed with which genome sequence data can be generated will be accompanied by an exponential increase in the number of sequenced eukaryotes. With the increasing number of sequenced eukaryotic genomes comes a need for bioinformatic techniques to aid in functional annotation. Ideally, genome context based techniques such as proximity, fusion, and phylogenetic profiling, which have been so successful in prokaryotes, could be utilized in eukaryotes. Here we explore the application of phylogenetic profiling, a method that exploits the evolutionary co-occurrence of genes in the assignment of functional linkages, to eukaryotic genomes.

**Results:**

In order to evaluate the performance of phylogenetic profiling in eukaryotes, we assessed the relative performance of commonly used profile construction techniques and genome compositions in predicting functional linkages in both prokaryotic and eukaryotic organisms. When predicting linkages in *E. coli *with a prokaryotic profile, the use of continuous values constructed from transformed BLAST bit-scores performed better than profiles composed of discretized E-values; the use of discretized E-values resulted in more accurate linkages when using *S. cerevisiae *as the query organism. Extending this analysis by incorporating several eukaryotic genomes in profiles containing a majority of prokaryotes resulted in similar overall accuracy, but with a surprising reduction in pathway diversity among the most significant linkages. Furthermore, the application of phylogenetic profiling using profiles composed of only eukaryotes resulted in the loss of the strong correlation between common KEGG pathway membership and profile similarity score. Profile construction methods, orthology definitions, ontology and domain complexity were explored as possible sources of the poor performance of eukaryotic profiles, but with no improvement in results.

**Conclusion:**

Given the current set of completely sequenced eukaryotic organisms, phylogenetic profiling using profiles generated from any of the commonly used techniques was found to yield extremely poor results. These findings imply genome-specific requirements for constructing functionally relevant phylogenetic profiles, and suggest that differences in the evolutionary history between different kingdoms might generally limit the usefulness of phylogenetic profiling in eukaryotes.

## Background

With the exponential growth rate of newly sequenced genomes, comparative genomics methods are increasingly important in providing frameworks of automated functional annotation for newly sequenced genomes. Approaches such as gene context, gene fusion [1-5], domain interactions [6], and phylogenetic profiling [7-13] have been used to help identify functional associations and assign putative roles for unannotated genes. In the past these comparative genomics methods have been applied primarily to prokaryotic genomes, in part due to the lack of sequenced eukaryotic genomes, and in part due to differences in genomic organization of eukaryotes. For example, gene context is of limited use in eukaryotes as the relationship between proximity of genes and functional relatedness is much weaker [14]. Despite fundamental differences between prokaryotes and eukaryotes, there is preliminary evidence that methods such as gene fusion and phylogenetic profiling may be viable techniques in the annotation of eukaryotic genes [9,15]. With the recent sequencing of more eukaryotic genomes, we are at a point where we can more thoroughly assess how useful comparative genomics methods may be in the annotation of eukaryotic genomes. Here we focus on phylogenetic profiling, a method of assigning functional associations based on the patterns of evolutionary co-occurrence of genes among many organisms. Our intent is to assess the ability to predict gene function in eukaryotic organisms based on patterns of phylogenetic conservation in different groups of organisms.

Genes with similar patterns of co-occurrence across many organisms tend to exist in the same protein complex, biochemical pathway or sub-cellular location [8,12]. The construction of profiles, which capture the phylogenetic distribution of the genes of a given organism, allows for the genome-wide identification of functional linkages between genes which themselves have limited known annotation [7]. The utility of this method is reflected in the success of previous studies, where putative associations have been shown to have a high reliability across a number of ontologies, for bacterial organisms as well as *S. cerevisiae *[8-11]. However, results in *S. cerevisiae *were obtained with profiles consisting of mostly prokaryotic organisms, limiting the predicted associations to those genes which are of microbial descent.

A phylogenetic profile of a gene is classically represented by a binary vector, representing the presence or absence of homologs to that gene across a set of organisms [7,8]. Presence or absence of homologs can be determined with orthology databases, such as COG [16], or by using raw sequence similarity scores, such as a BLAST [17] E-value, and imposing a threshold for presence. While manually curated orthology databases contain stringent definitions of common descent, they have lower coverage and suffer from infrequent updates due to limitations in manpower and an exponential growth in data. For these reasons, it is advantageous to be able to automate profile construction using only sequence similarity, which allows for greater coverage and the application of phylogenetic profiling to newly sequenced, unannotated organisms. In this vein, several methods have been developed which construct phylogenetic profiles from transformed BLAST E-values and bit scores. A comparison of commonly used methods does not currently exist in literature, so we initiated our analysis by performing this comparison in both prokaryotic and eukaryotic genomes. We hoped to provide insight for the community regarding which technique may be best when profiling prokaryotes. Furthermore, by assessing the predictive abilities in prokaryotes we established a benchmark to evaluate the quality of predicted association in eukaryotic genomes.

In addition to the method used to construct profiles, the set of organisms used is also of great importance. The genome composition of profiles was addressed recently by Sun, *et al *[11], who showed that accuracy of predictions for prokaryotic genomes was improved by using sets of genomes which are maximally distant from one another. In other words, using a more diverse set of organisms results in a more informative pattern of occurrence of a gene – possibly by reducing redundancy caused by having many closely related organisms. With our emphasis on annotating eukaryotic genomes, we sought to extend Sun's analysis of genome composition by analyzing the affect of eukaryotic genomes, both with and without additional prokaryotic genomes, on predicted associations. In virtually all previous studies, eukaryotes have been used in profile construction without an assessment of their influence on performance.

With the ultimate goal of assessing the prospects of phylogenetic profiling as a tool for identifying functional linkages in eukaryotic organisms, we explored the influence of both profile construction method and genome composition on performance in *E. coli *and *S. cerevisiae*. Our analysis of different profile construction methods reveals that in *E. coli *continuous profiles constructed from transformed BLAST bit scores perform best, while in *S. cerevisiae *discretized profiles have higher accuracy, based on common KEGG pathway membership of predicted associations. In addition, independent of the profile construction method used, in *S. cerevisiae *the addition of eukaryotic genomes to profiles consisting of a majority of prokaryotic organisms was extremely detrimental to performance; this was seen both in terms of the number of different KEGG pathways in which a nonrandom percentage of correct predictions are made, and the accuracy of these predictions within different pathways. Furthermore, when using profiles composed of strictly eukaryotic genomes, performance was drastically reduced based on all metrics used. Several attempts to account for potential reasons for this, such as greater domain complexity in eukaryotes, the uneven distribution of sequenced eukaryotes, and the functional biases of different ontologies (*e.g*. KEGG and GO) all failed to influence profiling performance. We conclude from these findings that fundamental differences between eukaryotic and prokaryotic evolution may restrict the usefulness of phylogenetic profiling in eukaryotes to a limited number of pathways.

## Results

### Comparison of existing methods of profile generation

Though an analysis of the effectiveness of applying phylogenetic profiling to eukaryotes has yet to be studied in literature, homology based methods of generating profiles for prokaryotes has been presented in numerous publications [7-9,11,13]. To initiate our study we investigated the relative performance of the commonly used previously documented profile generation methods in both *E. coli *and *S. cerevisiae*, using profiles consisting of only prokaryotic organisms. We wanted to establish a benchmark for subsequent predictions in eukaryotes, in addition to providing a side-by-side comparison of available methods to the benefit of the community, as currently none exists.

Existing homology-based profile creation methods can be placed into one of two classes: those that discretize their sequence similarity scores, and those that don't. Original phylogenetic profiles used a binary discretization method, where a gene was designated to be either absent (0) or present (1), based on the best hit BLASTP E-value into each organism being above or below a set cutoff [7,8]. Optimization of these binary profiles was recently explored by Sun and colleagues, where it was found that setting a cutoff for homolog presence at 10^-5 ^was optimal in *E. coli*, and this threshold was found to be robust when tested in other prokaryotes [11]. In this paper, we will refer to this optimal binary profile construction method as SM ("Sun Method"). Building on earlier work with binary profiles, Date and Marcotte presented a multinary discretization method where best hit BLASTP E-values were discretized into one of 11 bins; this was shown to be quite effective in both *E. coli *and *S. cerevisiae *[9]. In this paper we will refer to the Date and Marcotte method as DM ("Date Method"). Extending the DM method, in this paper we optimized the discretization process by identifying binning parameters which resulted in the maximal performance for a given organism, and refer to this method as SG ("Snitkin-Gustafson" method, see "Methods" for more details). SG was developed to determine the upper-bounds performance of using discretized BLAST scores by finding the query organism specific optimal binning parameters. Lastly, a method using continuous values was presented by Enault and colleagues, where normalized BLASTP bit scores are used [13]. This continuous profile method will be referred to as EM ("Enault Method").

**Figure 1:**
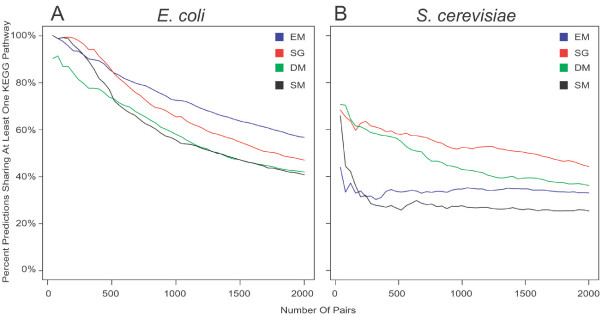
**Method performance comparison between SG, DM, EM and SM**. On the y-axis is the percent of predicted associations sharing at least one KEGG pathway, when looking at the N highest confidence predictions (x-axis). Four methods are compared: SM, a binary profile generated from BLAST E-values using 10^-5 ^as a cutoff for presence/absence; DM, a multinary profile that discretizes BLAST E-values into 11 equidistant bins; SG: a multinary profile that uses three bins in *E. coli *and 5 bins in *S. cerevisiae*, where presence/absence boundaries are optimally determined, and intermediate binning boundaries are equiprobable; EM, a profile consisting of normalized BLAST bit-scores. Profile similarity was measured for the SM, DM and SG methods using mutual information, while Pearson's correlation was used for the EM method. (A) *E. coli *and (B) *S. cerevisiae *are used as the query organisms, with SG in red, DM in green, SM in black and EM in blue. The profile genome composition used in both graphs is the Prok180 set, which is composed of entirely prokaryotic genomes.

To compare the performance of the different methods, we first used *E. coli *as the query organism against a reference organism set of 180 prokaryotes (Prok180). The KEGG pathway ontology was used to assess the accuracy of predicted associations. It should be noted that a few pathways were removed from consideration due to the lack of functional relationships among their members (see "Methods"). Figure 1A depicts the accuracy, as measured by positive predictive value (PPV), of the 2000 highest-confidence predictions made by each of the three published methods, and the SG method. The EM method, based on continuous profiles, has the best overall performance. We found that among the discretized methods, the SG method, using parameters optimized specifically for *E. coli *in order to identify the 'best case' accuracy for discretized phylogenetic profiling, had the highest accuracy. What is perhaps surprising is that DM, which uses multinary profiles that incorporate gradients of similarity, performed similarly to the binary SM method. This result, in conjunction with the better performance of SG, suggests that DM is losing information by discretizing into too many bins, thus making distinctions between E-values which in effect incorporate misinformation. In this respect using continuous profiles can be advantageous to any discretization method, as arbitrary cutoffs do not need to be imposed.

### Relative performance using a eukaryote as the query organism

A previous study has shown that when using *S. cerevisiae *as the query organism in conjunction with profiles composed of primarily prokaryotic organisms, there is a strong correlation between the profile similarity score and the functional relatedness of proteins [9]. Using Prok180, profiles were generated using all previously mentioned methods for *S. cerevisiae*. As seen in Figure 1B, the SG method, using parameters optimized specifically for yeast, outperforms the other three methods on this set. For all methods, the overall performance when using yeast as the query organism is lower than when using *E. coli*, as is expected due to its increased evolutionary distance to the organisms of which the profiles are composed.

To gain further insight into the predictions made by the methods and, specifically, to determine the diversity of the predictions made, we conducted a pathway centric analysis. This analysis makes use of the hypergeometric test and is described more thoroughly in "Methods." The purpose of this test is to determine the number of KEGG pathways in which the percentage of correct predicted associations is higher than random. This analysis was performed by looking at the top 2000 predictions for the methods with the highest accuracy among both method classes: SG representing the discretized profiles and EM representing the continuous profiles. When using *E. coli *as a query organism with the Prok180 set, a set of 48 pathways were found to be statistically enriched by SG, and 55 were enriched by EM. In contrast, when yeast was used as the query organism with same genome set, 34 pathways were found to be enriched by SG, and 24 by EM. It is unsurprising that fewer pathways were identified in *S. cerevisiae *when using a profile composed of solely prokaryotes; a large evolutionary distance to the profile organisms, coupled with eukaryotic specific functionality, limit the number of pathways which can be identified using the Prok180 set.

### Influence of the incorporation of eukaryotes among the reference organisms

A question that has gone unaddressed until recently is how the genomic composition of phylogenetic profiles affects performance in functional annotation. Many previous studies have taken the tack that all available sequenced genomes, prokaryotic and eukaryotic alike, should be included in the profile. Our findings strongly suggest two things about the creation of profiles in which the query organism is a eukaryote: first, having a significant number of eukaryotes in a profile, despite the presence of a majority of prokaryotes, will reduce the functional diversity of the results returned by the method; second, profiles consisting entirely of eukaryotes have very limited accuracy and coverage.

**Figure 2:**
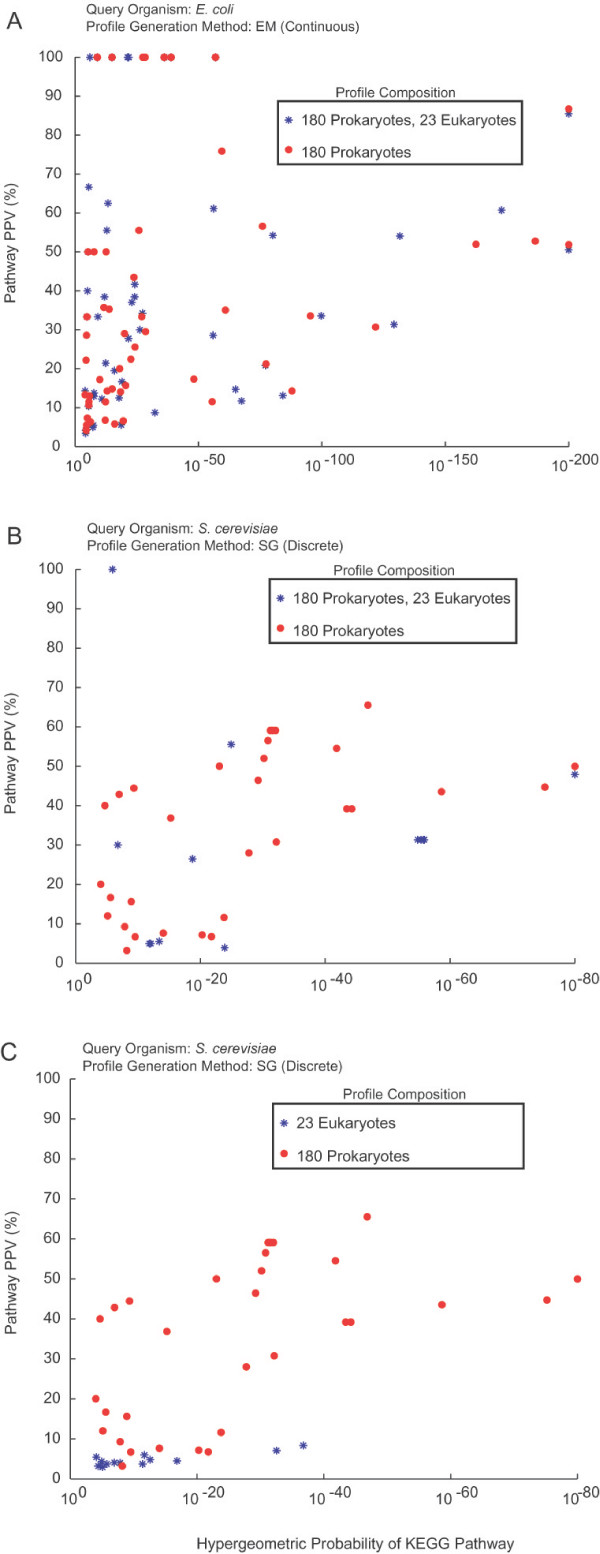
**Hypergeometric probability of KEGG pathways among high confidence predictions**. To determine which KEGG pathways have significant ratio of correct predictions among the highest confidence predictions, a hypergeometric probability analysis was performed. Each point on the graph represents a different KEGG pathway. On the x-axis is the hypergeometric probability that the pathway has more correct predictions than would be expected by chance, given the total number of predictions involving a member of the pathway. On the y-axis is the percentage of correct functional linkages involving members of the given pathway. By comparing the performance of profiles containing only prokaryotic organisms to profiles which also contain eukaryotes, it is evident that: (A) when *E. coli *was used as the query organism against Prok180 and Mix203, there is very little difference in performance. (B) When *S. cerevisiae *was used as query organism against Prok180 and Mix203, a large decrease in functional diversity, as measured by the number of significantly enriched KEGG pathways, was observed in the Mix203 set. Furthermore, there is also a marked decrease in accuracy even among those pathways which remain significantly enriched using the Mix203 set. (C) When *S. cerevisiae *was used as query organism against Euk23, while a small number of pathways were significantly enriched, they all had very poor accuracy, which greatly decreases the usefulness of their predictions as the false positives greatly outnumber the true positives.

Previous publications have used eukaryotic genomes in profile construction when available, and we wanted to determine the impact these genomes were having on the predicted associations. Twenty-three eukaryotic organisms were added to the Prok180 set, creating the set designated Mix203. First, a comparison of prediction accuracy using *S. cerevisiae *as a query organism was performed, showing a similar performance using the Mix203 set to that seen in Figure 1B for profiles constructed with Prok180. While overall accuracy remained comparable, the pathway centric analysis revealed some striking differences between predictions made using Prok180 and Mix203. In Mix203, when using yeast as the query organism, SG had 14 statistically enriched KEGG pathways, while EM had 12. Furthermore, the enriched pathways that remained significant tended to be housekeeping processes, such as ribosome, purine and pyrimidine metabolism, and proteasome. These markedly lower numbers of statistically enriched pathways, when compared to 34 and 23 in Prok180 for SG and EM, respectively, illustrate the loss of diversity that occurs with the introduction of eukaryotes into the Prok180 profile (see Figure 2B). This trend was also observed when looking at larger numbers of predictions for the various methods. The difference in the composition of the resulting associations can be partially explained by noticing that profile pairs present in high confidence predictions of Mix203, but not of Prok180, tended to be nearly ubiquitous in all the eukaryotic organisms. In essence, the addition of a string of identical bits into two previously weakly correlated profiles results in a much increased correlation. In other words, we observe correlations between genomes instead of between genes, typically produced among genes belonging to pathways ubiquitous to eukaryotes. The consequence of including eukaryotic organisms when identifying associations in *E. coli *is negligible based on both overall accuracy and pathway analysis, as shown in Figure 2A.

**Figure 3:**
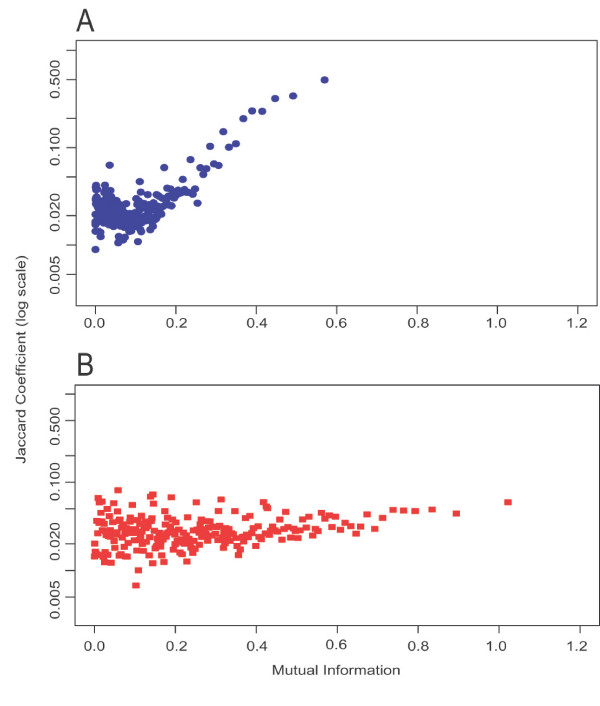
**Relationship between MI and Jaccard coefficient**. Mutual information (MI) computed using SG profiles plotted against Jaccard coefficient, based on KEGG pathway membership, using (A) Prok180 and (B) Euk23 profiles in *S. cerevisiae*. Predictions were ranked by MI values and the average of every thousand MI and Jaccard values was computed to represent a single point.

Even though the percentage of eukaryotic organisms making up the profile is only roughly 10% of total organisms, we still see a drastic change in performance in *S. cerevisiae*. One might expect *a priori *that using only eukaryotic organisms to construct profiles would be ideal when profiling a eukaryotic organism, but these results show no evidence of this effect. In order to test this explicitly, a new organism set was constructed that contained 23 eukaryotic organisms (Euk23). As can be seen in Figure 3, there is a much decreased correlation between profile similarity score and KEGG pathway similarity with Euk23 when using SG, when compared to the strong correlation using Prok180. The large decrease in performance in Euk23 is observed when using all profile creation methods (SG is shown in Figure 3B). In addition, calculation of the Jaccard coefficient using different levels of the Gene Ontology [18] instead of KEGG resulted in no improvement. The poor performance of the eukaryotic profile is also illustrated by the drastic decrease in number of pathways with significant hypergeometric probabilities, and the low accuracy of predictions even among the significant pathways (Figure 2C). These same trends were observed as well when using *E. coli *as the query organism against Euk23.

To ascertain whether this poor performance is a consequence of using homology-based profile generation, as opposed to the use of orthologous definitions, we created profiles using bi-directional best hits, which is a practice commonly used to define orthology [19,20]. No improvement in the correlation between profile similarity and various ontologies was observed when using profiles constructed in this manner. Another analysis we performed used profiles constructed from the KOG database [16], but using these profiles did not improve results either, although the small number of genomes (currently seven species) used in the construction of groups in KOG limits its statistical power for a profile similarity calculation.

### Possible sources of poor performance in eukaryotic profiling

We found that the poor performance in the Euk23 organism set is not an artifact of the choice of ontology, and is not circumvented by the use of orthologous gene definitions. Therefore we next explored possible evolutionary reasons for the lack of correlation (seen in Figure 3B) between profile similarity and pathway membership in eukaryotes. Two possible problems are the complex domain architecture of eukaryotic proteins, and insufficient diversity in the currently available sequenced eukaryotic genomes.

Using homology-based methods to generate phylogenetic profiles could lead to potential problems in eukaryotic genomes due to the promiscuity of some domains in proteins throughout these genomes. Specifically, there could be a significant BLAST hit into a genome against a protein which shares a common domain with the query, although a true ortholog is not present. By discretizing E-values to account for the degree of similarity it is hoped that some of these problems may be averted as true orthologs will likely receive more significant E-values than domain hits. But the potential for the presence of promiscuous domains to lead to spurious associations still exists, and in order to address this problem we tried limiting our analysis to proteins in *S. cerevisiae *which had zero or one Pfam [21] domains, as described in the methods section. The results show no increase in accuracy by looking strictly for associations among this filtered set. Another technique used to circumvent the problem of significant domain hits is to require that the BLAST alignment cover a predetermined percentage of the sequence of both the query and the target [22]. Application of this criterion did not affect the results (data not shown).

A second potential source of the poor results with using profiles composed of eukaryotes is the set of currently sequenced genomes. Although the number is large enough to obtain statistically significant associations, it is possible that the available genomes are too clustered on the evolutionary tree to create useful profiles. In other words, the significant associations detected are due to correlations between the genomes, and not between the genes. The existence of correlations in gene content has been discussed previously in literature, and in fact has been exploited in order to reconstruct phylogenetic trees [23-25]. Due to the small number of sequenced eukaryotes it is difficult to rule out genome composition as a source of error and a definitive solution will require a diverse set of eukaryotic genomes.

We attempted to get some indication as to whether or not the currently sequenced genome set is a problem by using a metric which we termed clade entropy (see "Methods"). Clade entropy is a weighted summation of the entropies within each clade, which we used to identify those profiles with high entropy due to selective pressure on the presence of the gene and not the relationship between genomes. We reasoned that if we eliminated profiles with low entropy within clades, we would avoid identifying spurious associations among all genes which may reside within a clade due to a close relationship among the constituent organisms. More specifically, there may be many independent processes which are clade specific, and interactions will indiscriminately be identified both within and between these processes. Examining significant relationships among only those profiles above various clade entropy cutoffs resulted in minimal improvement.

Although our attempts to account for inadequate genome diversity were unsuccessful, previous research utilizing the structure of the phylogenetic tree while making functional predictions indicates that this may be at least part of the problem [26]. Barker and Pagel performed an analysis in which linkages among yeast proteins were assigned p-values based on having a significantly correlated evolutionary pattern among a set of 15 eukaryotic genomes. Specifically, the number of times a pair of genes were gained and lost together based on the reconstructed phylogeny was shown to be predictive of functional relatedness. This suggests that profiles composed of eukaryotes may be informative, but the relationship among the constituent genomes convolutes the signal. The question still remains whether there is a genome set which will allow the less computationally demanding profile based methods to successfully predict functional linkages on a global scale.

## Discussion

The objective of this study was to assess the prospects of applying phylogenetic profiling to eukaryotic organisms. To initiate our study we assessed a number of currently used methods in both *E. coli *and *S. cerevisiae*, using profiles composed of prokaryotes. We showed that profiles composed of continuous bit scores perform better in *E. coli*, while discretized profiles are superior in *S. cerevisiae*. Overall performance is better in *E. coli *than in *S. cerevisiae*, which we attributed to the closer relationship of the profile organisms to *E. coli*. The better performance in *E. coli *was reflected in both the number of KEGG pathways in which a nonrandom percentage of correct functional linkages were predicted, and the prediction accuracy within those pathways. Surprisingly, the subsequent incorporation of eukaryotic organisms into the profiles results in a decline in pathway diversity in *S. cerevisiae*, calling into question the previously published incorporation of eukaryotic organisms into profiles, as has been standard.

We also explored the application of phylogenetic profiling using profiles consisting entirely of eukaryotes. Here, we found that there is a much decreased correlation between profile similarity score and common KEGG pathway membership, regardless of the profile generation method used. The use of more rigorous gene orthology definitions to create the profiles did not show any improvement in performance, nor did the use of different ontologies.

Based on a lack of improvement when examining associations between single domain proteins we ruled out domain complexity in eukaryotic organisms as the sole root of the eukaryotic profiling problem. Although this is not the only problem, it is likely that the power law distribution of domains in the proteins of eukaryotic organisms [27] will result in spurious hits when using homology based profiling. An alternative to accounting for the domain complexity in eukaryotes is to take advantage of the variable distribution of domains in eukaryotic organisms and perform profiling using domains instead of whole proteins. Given that domains are the functional units of proteins, identifying evolutionary dependencies between domains can yield more general insights into eukaryotic protein evolution. Profiling of individual domains in prokaryotic genomes has been implemented previously by Pagel et al. [28]. Its application to eukaryotes, with the caveat of using domain combinations, would be an interesting extension of this work.

A second potential issue with performing phylogenetic profiling on eukaryotic organisms is the lack of an effective set of sequenced eukaryotic genomes. Sun, *et al *[11], showed that there is an optimal set of organisms to be used when profiling prokaryotic organisms and this set has two defining characteristics. One is that organisms are maximally distant from each other, and the second is that the set is large. Neither of these criteria is met by the current set, which is relatively small and clustered on the evolutionary tree. Information theory suggests that in phylogenetic profiling the optimal signal is attained by using a diverse set of genomes having presence and absence of the genes of interest [10]. When the genomes are too clustered it is very difficult to distinguish correlations in the patterns of occurrence of genes from correlations among the genomes. Although we attempted to circumvent this problem using a metric to eliminate profiles which had limited diversity within different clades, we cannot rule out an uninformative set of currently sequenced eukaryotic genomes as a problem.

A final possibility, which cannot be addressed until more sequence data is available, is that evolution of eukaryotic genomes precludes the use of phylogenetic profiling on a genome-wide scale. There are several characteristics of prokaryotic genome evolution that make them suitable for phylogenetic profiling. First, functionally related cassettes of genes can be transferred as a unit between organisms, thereby directly maintaining their association in various genomes [29]. A second point related to this is that functionally linked genes are often located near each other in prokaryotic genomes. This fact is illustrated by the effectiveness of using patterns of chromosomal proximity to annotate genes [4]. Due to their chromosomal proximity, genes have an increased probability of being transferred or lost as a unit. The lack of these properties on a global scale in eukaryotic genomes may make the pattern of occurrence of genes less informative. In addition, the more complex regulatory framework in eukaryotes may put less of an importance on the presence of a gene and more on its temporal and spatial occurrence during cellular processes.

The utility of phylogenetic profiling in annotating eukaryotic genomes will not be known for certain until more sequence data becomes available. Even if the complexity of eukaryotic genomes makes profiling ineffective on a global scale, it is still likely to be a viable technique in the annotation of particular pathways. Previous studies have in fact identified uncharacterized participants in known biological processes by exploiting the knowledge of the phylogenetic distribution of the orthologs of known participants [30,31]. There are sure to be specialized pathways only present in subsets of organisms, and phylogenetic profiling can aid in the identification of their associated genes.

## Conclusion

With the rapid increase in newly sequenced organisms and the need for annotation, automated methods for functional prediction are essential. We focused our attention on the utility of phylogenetic profiling in predicting functional associations in eukaryotic organisms as this has gone largely unaddressed due to the minimal number of sequenced eukaryotic genomes previously available. Surprisingly, we found that the most effective organism composition for profiles when predicting functional associations in *S. cerevisiae *was one consisting solely of prokaryotes. We have discovered that despite their previous use in profile construction, the inclusion of eukaryotic organisms in profiles consisting of a majority of prokaryotes resulted in inferior performance based on the functional diversity of results. Furthermore, we have made an initial attempt to apply profiling with a wholly eukaryotic profile to a eukaryotic organism, and shown the results to be extremely poor. These findings have implications in both the optimal genome composition for phylogenetic profiling and indicating possible fundamental differences in the evolution of prokaryotic and eukaryotic genomes, which may limit the use of phylogenetic profiling in annotating eukaryotic organisms. At the very least our results indicate that caution should be used in the naïve application of context based methods, which have been tuned primarily in prokaryotes, to the annotation of eukaryotic genomes.

## Methods

### Data sets

252 completely sequenced organisms were downloaded from the Kyoto Encyclopedia of Genes and Genomes (KEGG) website [32]. For species with multiple strains sequenced, only a single one was used. Three subsets of reference organisms were used in this research:

1) Prokaryotic set (180 organisms) (Prok180): consisted of all proteins from prokaryotic organisms.

2) Eukaryotic set (23 organisms) (Euk23): consisted of all proteins from eukaryotic organisms. In addition to KEGG eukaryotic organisms, *G. lamblia*, *N. crassa*, *P. vivax *and *T. thermophila *were included [33-36].

3) Prokaryotic and Eukaryotic set (203 organisms) (Mix203): consisted of all proteins from the Prok180 and Euk23 data sets.

*E. coli *and *S. cerevisiae *were used as query organisms.

### Generation of phylogenetic profiles

For the creation of the optimally discretized profiles, which are referred to in this paper as SG, the following procedure was used. Amino acid sequences from the query organism were compared against each set of reference organisms using NCBI's BLASTP software [17]. Let *i *represent a protein in the query organism, and *j *represent a reference organism. Matrix *E *was created, such that the best BLAST E-value of protein *i *against reference organism *j *was stored in *E*_*ij*_.

To construct phylogenetic profiles *P*_*ij *_from *E*_*ij*_, two empirically-determined E-value thresholds were used. Let *T*_*a *_and *T*_*p *_be E-value thresholds for defining absence and presence, respectively, and let *N *be the total number of discrete values that the E-values will be discretized into.

*P*_*ij *_= 0 if *E*_*ij *_> *T*_*a*_

*P*_*ij *_= *N*-1 if *E*_*ij *_<= *T*_*p*_

Additional boundaries *T*_1 _to *T*_*N*-2 _are created, such that all *E*_*ij *_<*T*_*a *_and *E*_*ij *_> *T*_*p *_are divided into (*N*-2) equal-probable bins (with *P*_*ij *_= 1 to *N*-2). To generate equal-probable bins, a distribution was made of all E-values for all profiles not present in *T*_0 _or *T*_*N*-1 _(the bins for absence and presence, respectively), and boundaries were selected such that an equal number of E-values were in each bin. Thus, the E-values stored in *E*_*ij *_are converted to a discrete value, 0 to *N*-1, and stored in the *P*_*ij *_matrix.

E-value thresholds *T*_*a *_and *T*_*p *_were determined empirically for each of the query organisms. The empirically determined E-values for presence (the *T*_*p *_boundary) were selected from the set of 10^-5^, 10^-7^, 10^-10^, 10^-13^, 10^-15 ^and 10^-20^. An E-value was deemed optimal if it had the maximum number of correctly linked genes for the top 2000 predicted gene pairs, as ranked by mutual information. A correct linkage was defined by two genes sharing at least one KEGG pathway, though using GO annotation at various deep levels obtained similar results. This same procedure was used for the empirical determination of absence (the *T*_*a *_boundary), and number of bins (*N*). *T*_*a *_was empirically selected from the E-value set of 10^-1^, 10^-2 ^and 10^-3^. *N *was selected from the set of 3–10. For this study, *E. coli *was found to have optimal Tp, Ta and N values of 10^-5^, 10^-2 ^and 3; *S. cerevisiae *was found to have optimal Tp, Ta and N values of 10^-10^, 10^-2 ^and 5 when used against the Prok180 set (when used against Euk23 set, none of the parameters worked well).

To minimize the assignment of spurious linkages between housekeeping genes and lineage-specific genes, an overrepresented profile cutoff was used, where profiles seen more than 20 times were removed. For this filter, binary profiles for presence were defined by E-values < 10^-5^.

For the creation of phylogenetic profiles using the other methods compared here (DM, SM, EM), the procedures described in their respective papers were used [9,11,13].

### Assigning functional linkages

For the SG, DM and SM methods, mutual information (MI) was used to assess the correlation between two phylogenetic profiles. MI has previously been shown to have a high correlation with functional relatedness [9,10]. To compare profiles for the EM method, Pearson's correlation was used.

The Jaccard coefficient, a measure of pathway similarity, was used to measure the accuracy of the linkage assignments. Protein annotation from the KEGG Pathway Database was used to determine if a linkage assignment was predicted for proteins that existed in the same pathway. The Jaccard coefficient is measured as follows:

Jaccard Coefficient=NijNi+Nj
 MathType@MTEF@5@5@+=feaafiart1ev1aaatCvAUfKttLearuWrP9MDH5MBPbIqV92AaeXatLxBI9gBaebbnrfifHhDYfgasaacH8akY=wiFfYdH8Gipec8Eeeu0xXdbba9frFj0=OqFfea0dXdd9vqai=hGuQ8kuc9pgc9s8qqaq=dirpe0xb9q8qiLsFr0=vr0=vr0dc8meaabaqaciaacaGaaeqabaqabeGadaaakeaacqqGkbGscqqGHbqycqqGJbWycqqGJbWycqqGHbqycqqGYbGCcqqGKbazcqqGGaaicqqGdbWqcqqGVbWBcqqGLbqzcqqGMbGzcqqGMbGzcqqGPbqAcqqGJbWycqqGPbqAcqqGLbqzcqqGUbGBcqqG0baDcqGH9aqpdaWcaaqaaiabd6eaonaaBaaaleaacqWGPbqAcqWGQbGAaeqaaaGcbaGaemOta40aaSbaaSqaaiabdMgaPbqabaGccqGHRaWkcqWGobGtdaWgaaWcbaGaemOAaOgabeaaaaaaaa@506E@

Where *N*_*ij *_is the number of shared pathways between protein *i *and *j*, and *N*_*i *_and *N*_*j *_are the number of pathways that protein *i *and *j *are a member of, respectively.

Whole genome functional predictions for *E. coli *and *S. cerevisiae*, which include the MI score for the SG method, are available online [37].

### Analysis of enriched pathways

In order to gain deeper insight into the influence of the genome composition of profiles on predicted functional associations, beyond just cumulative accuracy, we performed a pathway centric analysis. In practice, functional associations predicted using phylogenetic profiling are likely to be used in reference to a particular pathway of interest. Therefore, in addition to the overall accuracy, the number of different pathways in which accurate associations can be made is a good indicator of the utility of the method in practice. In this vein we performed a hypergeometric probability analysis in order to assess the number of pathways in which the ratio of correct predictions to total predictions was greater than that expected by chance. In other words, this is a way of determining in which pathways phylogenetic profiling seems to be effective in identifying correct functional associations. The hypergeometric probabilities for each pathway were computed as follows:

P=(PCSC)(PT−PCST−SC)(PTST)
 MathType@MTEF@5@5@+=feaafiart1ev1aaatCvAUfKttLearuWrP9MDH5MBPbIqV92AaeXatLxBI9gBaebbnrfifHhDYfgasaacH8akY=wiFfYdH8Gipec8Eeeu0xXdbba9frFj0=OqFfea0dXdd9vqai=hGuQ8kuc9pgc9s8qqaq=dirpe0xb9q8qiLsFr0=vr0=vr0dc8meaabaqaciaacaGaaeqabaqabeGadaaakeaacqWGqbaucqGH9aqpdaWcaaqaamaabmGabaqbaeqabiqaaaqaaiabdcfaqnaaBaaaleaacqWGdbWqaeqaaaGcbaGaem4uam1aaSbaaSqaaiabdoeadbqabaaaaaGccaGLOaGaayzkaaWaaeWaceaafaqabeGabaaabaGaemiuaa1aaSbaaSqaaiabdsfaubqabaGccqGHsislcqWGqbaudaWgaaWcbaGaem4qameabeaaaOqaaiabdofatnaaBaaaleaacqWGubavaeqaaOGaeyOeI0Iaem4uam1aaSbaaSqaaiabdoeadbqabaaaaaGccaGLOaGaayzkaaaabaWaaeWaceaafaqabeGabaaabaGaemiuaa1aaSbaaSqaaiabdsfaubqabaaakeaacqWGtbWudaWgaaWcbaGaemivaqfabeaaaaaakiaawIcacaGLPaaaaaaaaa@499D@

P_T_: Total number of possible associations involving members of the pathway

S_T_: Total number of observed associations involving members of the pathway

P_C_: Total number of possible correct associations between members of the pathway

S_C_: Total number of observed correct associations between members of the pathway

Resulting p-values were Bonferroni corrected and an adjusted α of .01 was used as a cutoff to identify significant pathways.

### Clade entropy

The purpose of our clade entropy metric was to limit our analysis of profiles constructed using the Euk23 genome set to those whose variability is more likely to be indicative of functional selection of a gene. Our reasoning is that there are many genes which are specific to given clades, and among these genes many biological processes are represented leading to spurious hit between these processes. By removing these clade specific profiles we hoped to improve overall accuracy by eliminating spurious associations.

To compute clade entropy, the organisms in the Euk23 genome set were first assigned to one of four clades based on KEGG annotation (plants, animals, protists, fungi). Clade entropy (H_C_) was then computed as a weighted linear sum of the entropy within each clade, with weights being computed as the fraction of organisms represented by a given clade.

HC=∑i=0NC(NiNT)Hi
 MathType@MTEF@5@5@+=feaafiart1ev1aaatCvAUfKttLearuWrP9MDH5MBPbIqV92AaeXatLxBI9gBaebbnrfifHhDYfgasaacH8akY=wiFfYdH8Gipec8Eeeu0xXdbba9frFj0=OqFfea0dXdd9vqai=hGuQ8kuc9pgc9s8qqaq=dirpe0xb9q8qiLsFr0=vr0=vr0dc8meaabaqaciaacaGaaeqabaqabeGadaaakeaacqWGibasdaWgaaWcbaGaem4qameabeaakiabg2da9maaqahabaWaaeWaceaadaWcaaqaaiabd6eaonaaBaaaleaacqWGPbqAaeqaaaGcbaGaemOta40aaSbaaSqaaiabdsfaubqabaaaaaGccaGLOaGaayzkaaaaleaacqWGPbqAcqGH9aqpcqaIWaamaeaacqWGobGtdaWgaaadbaGaem4qameabeaaa0GaeyyeIuoakiabdIeainaaBaaaleaacqWGPbqAaeqaaaaa@4189@

N_i_: Total number of organisms in a given clade

N_T_: Total number of organisms in all clades (∑N_i_)

H_i_: Entropy within a given clade

N_C_: Total number of clades

H_C_: Clade entropy

Various cutoffs were then selected, at which only associations among profiles above that clade entropy cutoff were considered.

### Changes to KEGG annotation

While the KEGG pathway annotation was used to measure the performance of the different methods, we left out three pathways. These pathways were 2-component systems, ABC transporters and phosphotransferase systems. The reason these were left out is that the pathway maps contain many independent instances of these systems, and predicting associations between non-interacting members should not be rewarded [15]. This is important to note when using KEGG pathways for evaluation of protein-protein interaction predictions.

### Single domain proteins

HMMER was used to identify proteins in *S. cerevisiae *that do not contain multiple domains. After being trained on the Pfam-A and Pfam-B data sets, a subset of yeast proteins was created that contained <= 1 domain. This subset of proteins was then used as a query and run against the Euk23 reference organism set using the methods described above.

## Authors' contributions

ESS and AMG designed the study, performed the analysis and drafted the manuscript. JM, JW and CD conceived of the study and provided feedback throughout the project, and CD directed the work. All authors read and approved the final manuscript.
